# Synthesis, characterization of poly l(+) lactic acid and its application in sustained release of isosorbide dinitrate

**DOI:** 10.1038/s41598-024-56222-y

**Published:** 2024-03-25

**Authors:** El-Refaie Kenawy, A. M. Abd El.Hay, Nermeen Saad, Mohamed M. Azaam, Kamel R. Shoueir

**Affiliations:** 1 Polymer Research Group, Department of Chemistry, Faculty of Science, Tanta University, Tanta, 31527 Egypt; 2 Chemistry Department, Faculty of Science, Al Azhar University, Cairo, Egypt; 3 Regional Technical Manager, Pharma Business Group, IMCD, Cairo, Egypt; 4 Institute of Nanoscience and Nanotechnology, Kafrelsheikh University, Kafrelsheikh, 33516 Egypt

**Keywords:** Poly l(+) lactic acid, Polycondensation polymerization, Isosorbide dinitrate, Nanoencapsulation, Sustained release, Kinetics, Chemistry, Materials science

## Abstract

Poly l(+) lactic acid (PLLA) has become crucial in the biomedical industry for various uses. The direct polycondensation method was used to prepare Poly l(+) Lactic Acid (PLLA). Different catalysts, including metal oxides and metal halides, were used to test the polymerization technique. The effect of the amount of catalysts and the type of coupling agent were investigated. The effect of reaction time and polymerization solvents was also studied. PLLA was loaded with isosorbide dinitrate utilizing the solvent evaporation process. The synthesized polymer-drug system was evaluated by different means such as FT-IR, TGA, DSC, XRD, entrapment efficiency (E.E), drug loading (D.L), particle size analysis, and zeta potential determination. Studies on in-vitro release using UV light at 227 nm at various pH levels were conducted, and the kinetics of release and cytotoxicity using the sulforhodamine B (SRB) assay on human skin fibroblast cells were examined.

## Introduction

As an aliphatic polyester, poly l(+) lactic acid (PLLA) has become more important in the biomedical industry for a variety of uses, including bone fixation screws, suture threads, and drug delivery systems^1,2^. There are two primary pathways for synthesis. Condensation polymerization is the first, while ring opening polymerization (ROP) is the second^3,4^. Compared to ROP, direct polycondensation is a one-step technique that is more cost-effective^5^. The balance between free acids, water, and produced polyesters is the primary aspect of polycondensation that is impacted. The presence of water stops the process that would yield high molecular weight PLLA.

Water removal is necessary for the polycondensation process because the solution’s viscosity is reducing, and the polycondensation solvent makes removing water easier^6^. Previous research by Marques et al. demonstrated that utilizing high boiling point solvents made removing generated water easier^7^. A safe drug delivery system delivers drugs at specific locations, times, and concentrations^8,9^.

Isosorbide dinitrate relaxes vascular smooth muscle, causing it to dilate, especially the peripheral arteries and veins^10–12^. In medicine, isosorbide dinitrate has long been used to prevent and cure angina pectoris^12^. Isosorbide dinitrate has several dose-dependent adverse medication responses, including headache, weakness, moderate dizziness, sporadically changes in heart rate, nausea, vomiting, and sweating. Skin alterations brought on by isosorbide dinitrate include warmth, tingling, and redness^13^. However, when given orally, isosorbide dinitrate has a short half-life and low bioavailability (22–29%) due to substantial hepatic “first pass” metabolism, and the therapeutic efficacy is only present for a short time (t_1/2_ = 34–48 min)^14^. Both the oral mucosa and the gastrointestinal system absorb isosorbide dinitrate. In the liver, it is quickly metabolized. It takes 2–3 min to start working after sublingual administration and works for 1–2 h. After oral administration, the drug begins to work within 15–30 min, lasting around 4 h^15^.

Isosorbide dinitrate are widely used as therapeutic agents in the treatment of coronary heart disease, not only in patients with stable angina pectoris, but also in those with unstable angina pectoris, acute myocardial infarction and heart failure^16^. There have been claims to be numerous regulated delivery systems for the long-acting effects of isosorbide dinitrate^17^. To prevent pre-systemic elimination when given orally, Nozaki et al. created mucoadhesive transmucosal therapeutic systems (TmTs) using PVP Ks^18^. Bioadhesive polymer-grafted starch microspheres for sustained ISDN release in buccal administration were created by Vyas and Jain^15^. Emulsification-solvent evaporation was used by Dinarvand et al. to produce a slow-release formulation based on ethyl cellulose matrix microspheres^19^.

Unfortunately, the full realization of combination therapy through systemic delivery of free drugs is hampered by challenges such as poor solubility, variable stability, unsynchronized pharmacokinetics, and uneven tumor accumulation of combination drugs. The application of nanoparticles for simultaneous drug delivery represents a promising solution to overcome these obstacles^20–22^. This approach aims to overcome the drug efflux associated with extrinsic resistance, increase the circulation half-life in the bloodstream, facilitate drug targeting through surface functionalization and enable spatio-temporally controlled release of the drug cargo^23^. The formulation of nanoparticles using PLA as drug carrier shows remarkable improvements manifested in sustained release pattern and robust protection of the encapsulated drug from degradation over a prolonged period of time^3,24^.

The objectives of the current work are to prepare PLLA with different weight fractions using the direct polycondensation technique, which is a practical and affordable method, and to explore the variables that affect the polycondensation polymerization of PLLA, including the types and amounts of catalysts and coupling agents, solvents, reaction times, and solvent ratios. Nanoparticles of PLLA and the drug are then prepared by solvent evaporation to improve the release of the drug and avoid overdosing and adverse effects. Finally, investigate the cytotoxicity of human skin fibroblast cells determined by SRB assay and the release kinetics of isosorbide dinitrate from PLLA nanoparticles.

## Materials and methods

### Materials

A generous donation of isosorbide dinitrate (ISDN, USP, 40 mg) was provided by the EIPICO Company in Cairo, Egypt, bioavailability of ISDN is approximately 25%. *P*-Toluene Sulphonic Acid, Maleic anhydride, Zinc chloride, Calcium chloride, zinc oxide, xylene and diethyl ether from Alpha Chemika (India) and magnesium oxide from BDH laboratory supplies in Poole, BH151TD (England). From BIO CHEM (Egypt), Polyvinyl alcohol (M.wt 10.000), decalin and chloroform (assay 99%), pure ethyl alcohol (assay 99.9%), Ferric oxide, Magnesium chloride, Arsen (III) oxide, Acetone, Magnesium metal, Phthalic anhydride, Tin oxide, Antimony oxide, potassium hydroxide, phenphethalin, and stannous chloride dihydrate. Calcium oxide and l(+) lactic acid (assay 88–92%) were obtained from LOBA CHEMIE (India). Aluminum oxide was purchased from RIEDEL–DE HAEN AG SEELZE-HANNOVER (China). Zinc metal, tween 80, anhydrous hydrogen phosphate, Potassium dihydrogen orthophosphate, and Sodium chloride were purchased from ADWIC (Egypt). Pluronic F88 and Tetronic 1307 were gifted from BASF, USA. Without additional purification, all substances were utilized precisely as they were given.

### Characterization

#### FT-IR spectroscopy

Burker’s TENSOR 27-series FT-IR measurements in the 400–4000 cm^-1^ range using KBr pellets to define the functional group structure of samples using chloroform as the solvent.

#### ^1^H nuclear and ^13^C nuclear magnetic resonance

^1^HNMR and ^13^CNMR spectra using CDCl_3_ on a Bruker Avance II spectrometer running at 400 MHZ were given in ppm to illustrate the chemical configuration and the chemical shifts (δ).

#### Molecular weight measurements

An Alliance GPCV 2000 (Waters, US) was used to calculate the average molecular weight (Mw) at 30 °C. The mobile phase, tetrahydrofuran (THF), was set at a 1 mL/min flow rate. The polystyrene standards were used to produce the calibration curve. By using gel permeation chromatography (GPC), the Mw and molecular weight distribution index (Mw/Mn) was calculated. The Shodex DEGAS KT-16 de-gasser, the Shodex RI SE-31 RI detector, the Shimadzu C-R7A Chromatopac data processor, and the Shimadzu LC-10A pump made up the analyzer. Chloroform was utilized as the eluent, and two polystyrene gel columns-the Toso TSK gel G4000H8 and G2500H8-were combined.

#### Viscosity measurement

The measurement of viscosity was conducted by employing Schott Geräte’s Cannon–Fenske viscometers, which had a k constant of 0.002061 and a type of 520 00.

At a temperature of 25 °C, the Solomon-Cuita equation was utilized to determine the intrinsic viscosity [η] in chloroform (with a concentration of 2 g/dl) through a single-point evaluation^25^.

1 $$\left[ \eta \right] = \frac{{\sqrt {2(\eta sp - \ln \eta r)} }}{C}$$

where η_r_ = t/t_0_, η_sp_ = η_r_-1are the relative and the specific viscosity, respectively. c is the concentration of PLLA.

Additionally, the Mark–Houwink–Sakurada relation was used to compute the viscosity molecular weight (Mv) of PLLA, as shown follow^26^.

2 $$\left[ \eta \right] = KM^{\alpha }$$

K = 1.29 × 10^–4^, α = 0.82 (for PLA)^27^.

#### Thermal gravimetric analysis (TGA)

Thermal stability, maximum degradation temperature, and change in mass with temperature were all measured for the samples using TGA analysis. Using a thermal analyzer Perkin Elmer 4000 and a heating rate of 10.0 degrees per minute between 50 and 800 °C. The temperature (T_onset_) could be estimated at which thermal degradation started and how the mass changed as the temperature rose. To determine the highest degradation temperature, derived thermogravimetric curves (DrTG) were employed (Tdeg._max_.)^28^.

#### Differential scanning calorimetry (DSC)

Polymeric materials are frequently examined using DSC thermograms to identify their thermal transitions. The glass transition temperature (Tg), crystalline temperature (Tc), and melting temperature (Tm) are significant thermal transitions. A Shimadzu DSC60 (Japan) was used to measure the thermal characteristics of the produced polymers at a heating rate of 10 C/min.

#### X-ray diffraction (XRD)

X-ray pattern was determined using an XMD 300 (Unisantis, Germany). Cu-K radiation, with a wavelength of 1.5406, was run at 45 kV and 0.8 mA with an angle range of 5 to 90, and the scanning rate was set at 0.05/sec.

#### The particle size & zeta potential measurements

Zetasizer Nano ZS, Malvern, UK, was used to measure the particle.

#### Scanning electron microscopy (SEM)

The surface morphology of the nanoparticles was observed by SEM analysis using a SU8000 20 kV 4.0 mm X 25.0 K SE(U). Briefly, a thin layer of gold was sputtered onto the particles to facilitate visualization in the SEM. Images were acquired with an electron beam acceleration voltage of 15–20 kV.

### Synthesis

#### Synthesis of PLLA

In a round flask fitted with a Dean Starck apparatus and the appropriate amounts of catalyst and coupling agent, 30 mL of xylene and/or a mixture of xylene and decalin were added to 0.1 mol of l-(+) lactic acid^6^. The reaction mixture was agitated at a reflux temperature of 250 °C for 10 to 25 h. Filtering was done on the resultant PLLA. To eliminate the catalyst that hadn’t dissolved, PLLA was first dissolved in chloroform. The precipitation of the polymer solution in chloroform was achieved by introducing diethyl ether. A solid or semi-solid product was produced using a rotary evaporator under a vacuum to extract the solvents. The item was cleaned with 100% ethanol before being dried in a vacuum oven at 30 °C.

#### Effect of solvent types and reaction time

PLLA was polycondensed using two different types of solvents; Xylene alone or in combination with Decalin (1:1) and stannous chloride dihydrate as a catalyst (75 mg). The effect of reaction time was investigated by experimenting twice, once for 10 h and the second for 25 h, with (magnesium oxide 75 mg) as a catalyst.

#### Effect of catalyst types and their quantity

Magnesium oxide catalyst in varying concentrations was used to study the effects of catalyst type and quantity (50 mg and 75 mg). In addition, different types of catalysts, including magnesium oxide, stannous chloride dihydrate, calcium oxide, aluminum oxide, ferric oxide, zinc chloride, zinc metal, zinc oxide, calcium chloride, antimony trioxide, Arsen III oxide, titanium oxide, strontium chloride, and magnesium metal, were used to investigate the type of catalyst used in the polymerization of PLLA.

#### Effect of coupling agent types and catalyst/coupling agent ratio in polycondensation of PLLA

To polymerize PLLA, various coupling agents, including *p*-toluene sulphonic acid, maleic anhydride, and phenyl maleate^29^, were combined with three different catalysts: ZnO, MgO, and ZnCl_2_ according to illustrated in Tables 1, 2, 3 and 4.

**Table 1:** Effect of the ratio between MgO and different coupling in polycondensation of PLLA.

Reaction No.	Type of coupling agent	The ratio of MgO/coupling agent
1	*P-*toluene sulphonic acid	1:1
2	*P-*toluene sulphonic acid	2:1
3	*P-*toluene sulphonic acid	3:1
4	Maleic anhydride	1:1
5	Maleic anhydride	2:1
6	Maleic anhydride	3:1

**Table 2:** Effect of the ratio between ZnO and different coupling agents.

Reaction No.	Type of coupling agent	The ratio of ZnO/coupling Agent
1	*P*-toluene sulphonic acid	1:1
2	*P*-toluene sulphonic acid	2:1
3	*P*-toluene sulphonic acid	1:2
4	*P*-toluene sulphonic acid	1:3
5	*P*-toluene sulphonic acid	3:1
6	*P*-toluene sulphonic acid	2:2
7	Maleic anhydride	2:2
8	Maleic anhydride	1:1
9	Maleic anhydride	2:1
10	Maleic anhydride	3:1
11	Phethalic anhydride	2:2
12	Phethalic anhydride	1:1
13	Phethalic anhydride	2:1
14	Phethalic anhydride	3:1

**Table 3:** Effect of type of coupling agents with ZnCl_2_.

Reaction No.	Type of coupling agent (1:1)
1	*P*-TSA
2	Maleic anhydride
3	Phthalic anhydride

**Table 4:** Effect of change between catalyst and coupling agent.

Reaction No.	Catalyst	Coupling agent	The ratio between the catalyst and coupling agent
1	ZnO	ZnCl_2_	2:2
2	ZnCl_2_	ZnO	2:2

#### Acid value determination (AV)

25 mL of acetone was used to dissolve the material in a conical flask with a volume of 250 mL, ranging from 0.2 to 0.5 g. After a five-minute stand-by, phenolphthalein was used as an indicator to titrate the solution with an alcoholic potassium hydroxide solution of 0.1 N. A simultaneous blank analysis without a sample was carried out. Equation (3) denotes the acid value formula that indicates the quantity of potassium hydroxide required to neutralize 1 g of the sample.

3 $${\text{Acid}}\;{\text{value}} = \left( {{56}.{1}\left( {{\text{A}} - {\text{B}}} \right)*{\text{N}}} \right)/{\text{W}}$$

N is the KOH solution’s normality, W is the sample’s weight, and A and B are the amounts of KOH solution consumed by the sample and the blank reading, respectively (g)^27^.

#### Encapsulation of isosorbide dinitrate in PLLA nanoparticles

Solvent evaporation was the method used in this synthesis. PLLA was dissolved in a 7:1 mixture of dichloromethane and diethyl ether; then, isosorbide dinitrate was added while stirring for an hour to the polymer solution to achieve total miscibility. The surfactant solution in distilled water was combined with the previous drug-containing solution. The mixture was ultrasonically processed for 15 min while being swirled at 700 rpm and kept at 25 °C. After stirring for 15 min, the resultant solution was added to 40 mL of distilled water at 70 °C. When the temperature was raised to (40–45 °C), the solvent vaporized quickly, creating nanoparticles. The mixture was centrifuged at 6000 rpm for 30 min to remove the supernatant water, which was then cooled to room temperature. The remaining nanoparticles were rinsed three times with distilled water before being subjected to lyophilization.

#### Effect of surfactant type on the nanoparticles size and entrapment efficiency

Several tests were conducted with constant molecular weights of PLLA, constant ratios between polymer and drug, constant surfactant concentrations, and the addition of several types of surfactants to examine the impact of surfactant type on the nanoparticle size and entrapment efficiency. The experiment’s conditions are listed in Table 5.

**Table 5:** The effect of surfactant types.

Formula No.	PLLA M.wt	PLLA concentration (%)	Drug concentration (%)	Surfactant concentration (%)	Type of surfactant
F1	11,636	15	15	0.2	Pluronic F88
F2	11,636	15	15	0.2	Tetronic 1307
F3	11,636	15	15	0.2	Tween 80
F4	11,636	15	15	0.2	Polyvinyl alcohol

#### The effect of the ratio between PLLA and isosorbide nitrate on the nanoparticles size and entrapment efficiency

Several tests were conducted utilizing constant PLLA molecular weights, various polymer and drug ratios, constant surfactant concentrations, and Pluronic F88 as a surfactant to examine the impact of the ratio between PLLA and isosorbide on the nanoparticle size and entrapment efficiency. The experiment’s conditions are listed in Table 6.

**Table 6:** The effect of the ratio between PLLA and isosorbide nitrate.

Formula No.	PLLA M.wt	PLLA concentration (%)	Drug concentration (%)	Surfactant concentration (%)	Type of surfactant
F5	11,636	15	30	0.2	Pluronic F88
F6	11,636	30	15	0.2	Pluronic F88

#### Effect of molecular weight of PLLA on the nanoparticles size and entrapment efficiency

Various tests employing varied molecular weights of PLLA, a 2:1 ratio between PLLA and drug, constant surfactant concentrations, and Pluronic F88 as a surfactant were conducted to examine the impact of PLLA’s molecular weight on the nanoparticle size and entrapment efficiency. The experiment conditions are listed in Table 7.

**Table 7:** The effect of molecular weight of PLLA.

Formula No.	PLLA M.wt	PLLA concentration (%)	Drug concentration (%)	Surfactant concentration (%)	Type of surfactant
F7	24,165	15	30	0.2	Pluronic F88
F8	11,051	15	30	0.2	Pluronic F88
F9	11,239	15	30	0.2	Pluronic F88

### Determination of encapsulation efficiency (E.E)

The UV–visible spectrometer (9IS80-7000-10 Libra S60PC) was used to test the drug entrapment and encapsulation effectiveness^30,31^.

4 $${\text{lab}}.entrapment\;efficiency = \frac{{{\text{mass}}\;{\text{of}}\;{\text{drug}}\;{\text{encapsulated}}}}{{{\text{mass}}\;{\text{of}}\;{\text{the}}\;{\text{initial}}\;{\text{drug}}}} \times 100$$

5 $$mass\;loading\;capacity = \frac{{{\text{mass}}\;{\text{of}}\;{\text{drug}}\;{\text{encapsulated}}}}{{{\text{mass}}\;{\text{of}}\;{\text{the}}\;{\text{polymer}}}} \times 100$$

Using a UV/Vis spectrophotometer to scan an isosorbide dinitrate solution in ethanol, the maximum wavelength detected at 227 nm. To determine entrapment effectiveness, drug loading capacity, and for in vitro release experiments, different concentrations of the drug solution were generated, absorbance was measured, and a calibration curve was created.

### In vitro drug release

The behavior of the release of isosorbide dinitrate was investigated using the dialysis technique. In summary, a dialysis membrane with a molecular weight cutoff of 1000 KDa was used. 100 mg of the prepared nanoparticles were dissolved in 3 mL of phosphate buffer and placed in a dialysis bag. The bag was immersed in 60 mL of phosphate buffer (pH 7.4, 6.7, 4.5 and 1.2) and gently shaken at regular intervals at 37 °C and 80 rpm. Then two samples of 2 mL each were taken and measured with a UV spectrophotometer at a wavelength of 227 nm.

#### Cytotoxicity assay

Cell viability was determined by means of the SRB test. After preparing a cell suspension of 5 × 10^–3^ cells, 100 μL aliquots were cultured in full medium for a duration of 24 h using 96-well plates. A fresh batch of 100 L of solution with varying amounts of drugs was administered to the cells for treatment. The cells were rendered stable by replacing the solution with 150 μL of 10% TCA and keeping them at 4 °C for an hour following 72 h of drug exposure. Following the removal of the TCA solution, the cells were rinsed with distilled water on five separate occasions. After placing aliquots of a solution containing SRB in a 70 μL container, the mixture was kept in a dark room at room temperature for 10 min. The dishes were washed thrice using a solution of 1% acetic acid and left to dry in the air for the night. After dissolving the SRB stain that was bound to the protein, 10 mM TRIS was added in 150 μL quantity. The absorbance was measured at 540 nm using a microplate reader called BMG LABTECH^®^-FLUOstar Omega (Ortenberg, Germany)^32^.

## Results and discussion

According to the procedures outlined in the experimental section, PLLA was synthesized. L-Lactic acid was polycondensed to produce the polymerization utilizing a variety of catalysts. The creation of cyclic by dimer l-Lactic acid is the initial step. The latter was broken down into PLLA by using various coupling agents. The yield ranged from 35 to 90%. The solubility of the drug and polymer was considered when developing the solvent evaporation method to create PLLA/isosorbide dinitrate nanoparticles.

### FT-IR spectroscopy

**Figure 1:**
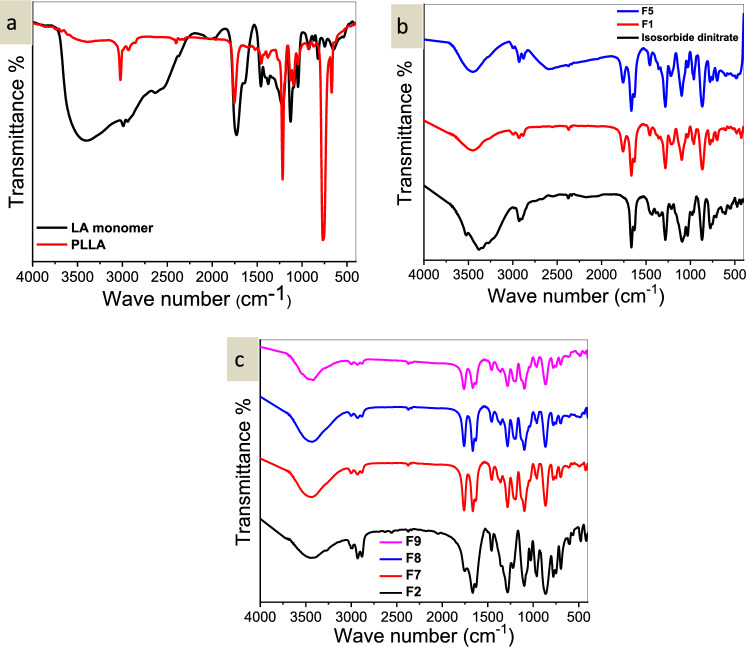
(**a**) FT-IR of LA monomer and PLLA, (**b**) FT-IR of isosorbide dinitrate, F1, and F5 and (**c**) of F2, F7, F8, F9.

Typical ester absorption peaks were observed in the PLLA FT-IR spectrum at 1761 cm^−1^ for the –COO– and 1092 cm^−1^ for the –O–, and at 2948–2998 cm^−1^ for the –CH_2_– and CH_3_ groups. As lactic acid polymerizes, the hydroxyl group bonds with the carboxyl group of another molecule, weakening the significant –OH stretch peak at 3405 cm^−1^. The hydroxyl absorption peak almost disappeared in PLA, indicating a decrease in the number of hydroxyl groups, while a larger C = O stretching absorption peak (1761 cm^−1^) was seen during subsequent production steps^33^. Absorption at 870.8 cm^−1^ indicates the amorphous phase of PLLA, while the crystalline phase is indicated by the band at 755.7 cm^−1^ (Fig. 1a). Similar outcomes were previously attained using the technique outlined by J. Diani and K. Gall^34^.

Isosorbide dinitrate’s FT-TR spectra reveal bands (2950–2850 cm^−1^) associated with aliphatic C–H stretching, bands at 1665–1635 cm^−1^ for asymmetric NO_2_ stretching, a band at 1460 cm^−1^ for methylene scissoring, at 1100 cm^−1^ due to asymmetric C–O–C stretching, and at 865 cm^−1^ for O–NO_2_ stretching (Fig. 1b). This agrees with the drug’s reported process data^35^.

The FT-IR spectra of the encapsulated isosorbide dinitrate showed that the polymer and pure drug maintained their distinct peaks. This indicates that there was no chemical reaction between the drug and polymer during the nanoencapsulation process. Furthermore, the chemical structures of the drug remained unchanged and did not undergo any degradation. The spectral analysis of PLLA/isosorbide dinitrate nanoparticles did not reveal any significant shift or loss of functional group peaks, indicating the compatibility between PLLA and Isosorbide dinitrate drug (Fig. 1a–c).

### ^1^HNMR spectra

Various proton resonances were identified in the ^1^H NMR spectra, which further elucidate the molecular structure of the synthesized PLLA. Methyl proton resonances were detected at 1.57 ppm, while methane proton resonances appeared at 5.15 ppm within the main chain. In addition, a subtle signal at 4.30 ppm and 1.4 ppm was assigned to the methine proton and the methyl proton near the terminal hydroxyl and carboxyl groups, respectively. The distinct peaks in the spectra confirm the successful preparation of PLLA and are in agreement with similar results obtained by Choubisa et al.^36^ who used a solid acid catalyst system for the polycondensation process. The results obtained are in agreement with previous studies where different catalysts were used and where ^1^H NMR analysis served as a central tool to characterize the resulting polymers. A comprehensive summary of the spectroscopic data can be found in Table 8 for clarity.

**Table 8:** ^1^HNMR peaks in each sample of prepared PLLA.

Catalyst used	CH ppm	CH_3_ ppm	CH(OH) ppm
ZnO	5.209	1.621	4.402
ZnCl_2_	5.208	1.621	4.406
ZnO/ZnCl_2_ (2:2)	5.262	1.625	4.406
Sb_2_O_3_	5.241	1.606	4.379
SnCl_2_	5.282	1.607	4.400
MgO/MA (2:1)	5.241	1.605	4.389

### ^13^C NMR

The ^13^C NMR spectra of PLLA show three distinct peaks at 16.59 ppm, 68.98 ppm and 169.56 ppm, respectively (Table 9). These peaks can be assigned to specific carbon atoms within the PLLA structure, namely the methyl, methane and carbonyl carbons. The resonance positions of these peaks provide valuable insight into the molecular arrangement of PLLA. The observed peaks are in agreement with the results of G. X. Chen et al. who used a similar method for the direct polycondensation of L-lactic acid to synthesize PLLA^37^. This agreement of the results underlines the consistency and reproducibility of the synthesis process and supports the reliability of our PLLA characterization.

**Table 9:** ^13^CNMR peaks in each sample of prepared PLLA.

Catalyst used	CH ppm	CH_3_ ppm	CH(OH) ppm
ZnO	69.44	16.72	169.59
ZnCl_2_	69.44	16.72	169.67
ZnO/ZnCl_2_ (2:2)	69.44	16.72	169.66
Sb_2_O_3_	69.43	16.72	169.58
SnCl_2_	69.43	16.73	169.59
MgO/MA (2:1)	69.44	16.72	169.60

### Molecular weight analysis and acid value determination

#### Effect of solvent

By employing stannous chloride as a catalyst and xylene as a solvent, PLLA was polymerized to determine the impact of the solvent on the molecular weight of the polymer. Another experiment was run using a 1:1 combination of Xylene and Decaline. For instance, when xylene was used, the PLLA M.wt was 10,099 Dalton, but when xylene and decalin were combined, the PLLA M.wt was 24,874 Dalton. The findings demonstrated that PLLA created using a solvent mixture yielded a greater M.wt than with xylene alone, primarily because Decalin has a higher boiling point than xylene, which facilitates the polycondensation reaction.

#### Effect of reaction time

By running the reaction for 10 and 25 h with MgO as a catalyst, it was possible to examine the impact of polymerization duration on the M.wt of the resulting PLLA. The response time was lengthened, which enhanced the M.wt of PLLA.

#### Effect of type of catalyst

Different metal oxides and metal chlorides were used as catalysts to study the impact of catalyst type on the polymerization of PLLA. The M.wt 25,114 Dalton yield of antimony trioxide was the highest, according to the results. Table 10 summarizes these data.

**Table 10:** The molecular weight data and an acid value of prepared PLLA samples.

Catalyst	Time (h)	Mw	[η]	Mv	AV
MgO 75 mg	10	3018	0.2121	3113	24.08
MgO 75 mg	25	8480	0.2202	8747	28.05
MgO 50 mg	25	1554	0.055	1603.6	56.1
SnCl_2_ (xylene only)	25	10,099	0.2541	10,416.8	56.1
SnCl_2_ (Xylene: Decalin)	25	24,874	0.5510	26,766	14
CaO	25	4045	0.1200	4172.5	28.05
Al_2_O_3_	25	6557	0.1783	6763.4	37.4
Fe_2_O_3_	25	11,590	0.285	11,955	28.05
ZnCl_2_	25	11,051	0.265	10,974	14
Zn metal	25	6754	0.1827	6967	14
ZnO	25	11,356	0.4135	18,858	14
CaCl_2_	25	2979	0.0934	3073.3	42.08
Sb_2_O_3_	25	25,114	0.6482	32,625.3	14
As_2_O_3_	25	3823	0.1146	3944.1	97.6
TiO_2_	25	2788	0.0885	2876.3	105.2
Mg metal	25	1111	0.0535	1550.7	168.3
Strontium chloride	25	4004	0.119	4130	112.2
MgO/*p*-TSA (1:1)	25	3803	0.1141	3923	80.1
MgO/*p*-TSA (2:1)	25	6186	0.1700	6381	28.05
MgO/*p*-TSA (3:1)	25	2227	0.0736	2298	35.06
MgO/MA (1:1)	25	4166	0.1229	4298	30.05
MgO/MA (2:1)	25	11,636	0.246	10,007	28.05
MgO/MA (3:1)	25	5286	0.1495	5453	65.1
ZnO/*p*-TSA (1:1)	25	1745	0.0602	1800	70.1
ZnO/*p*-TSA (2:1)	25	11,239	0.2163	8559	14
ZnO/*p*-TSA (1:2)	25	5948	0.1647	6136	14
ZnO/*p*-TSA (1:3)	25	9006	0.2314	9290	42.08
ZnO/*p*-TSA (3:1)	25	5033	0.1436	5192	33.0
ZnO/*p*-TSA (2:2)	25	10,169	0.2556	10,489	28.05
ZnO/MA (2:2)	25	595	0.0249	614.6	84.2
ZnO/MA (1:1)	25	983	0.1308	1014	33.0
ZnO/MA (2:1)	25	9208	0.3263	14,129	70.1
ZnO/MA (3:1)	25	7823	0.2061	8069	37.4
ZnO/PA (2:2)	25	3964	0.1180	4089	80.1
ZnO/PA (1:1)	25	6403	0.1749	6605	112.2
ZnO/PA (2:1)	25	2731	0.0869	2817	168.3
ZnO/PA (3:1)	25	8297	0.2163	8559	74.8
ZnCl_2_/*p*-TSA	25	2617	0.0840	2700	93.5
ZnCl_2_/MA	25	1304	0.0580	1719	112.2
ZnCl_2_/PA	25	1975	0.0667	2038	112.2
ZnO/ ZnCl_2_ (2:2)	25	10,316	0.2701	11,218	61.1
ZnCl_2_/ZnO(2:2)	25	2192	0.0726	2261.7	43.3

Mw, Molecular weight average; [η], intrinsic viscosity; AV, acid value; Mv, molecular weight measured by viscosity.

#### Effect of amount of catalyst

By performing the reaction with 50 mg and 75 mg MgO, it was possible to compare the effects of the two amounts of catalyst on the polymerization of PLLA. According to the data, M.wt produced 8480 Dalton when 75 mg of MgO was used, but M.wt produced 1554 PLLA when 50 mg of MgO was used. The degree of polymerization increased as the catalyst concentration did as well. Table 10 explains this information.

#### Effect of the ratio between catalyst and coupling agent

The researchers investigated the effects of the relationship between catalyst and coupling agent. In the case of MgO as catalyst and *p*-toluene sulfonic acid as coupling agent, the results of the experiments showed that a ratio of 2:1 between catalyst and coupling agent gave the best results. This ratio was supported by the high molecular weight produced in this example, which was higher than ratios of 1:1 or 3:1, indicating that a higher catalyst ratio is associated with a higher degree of polymerization. However, the molecular weight of the polymer produced decreased when the catalyst was increased to level 3. The molecular weight increased when maleic anhydride was replaced by *p*-toluene sulfonic acid at a ratio of 2:1 as a coupling agent (Table 10).

**Figure 2:**
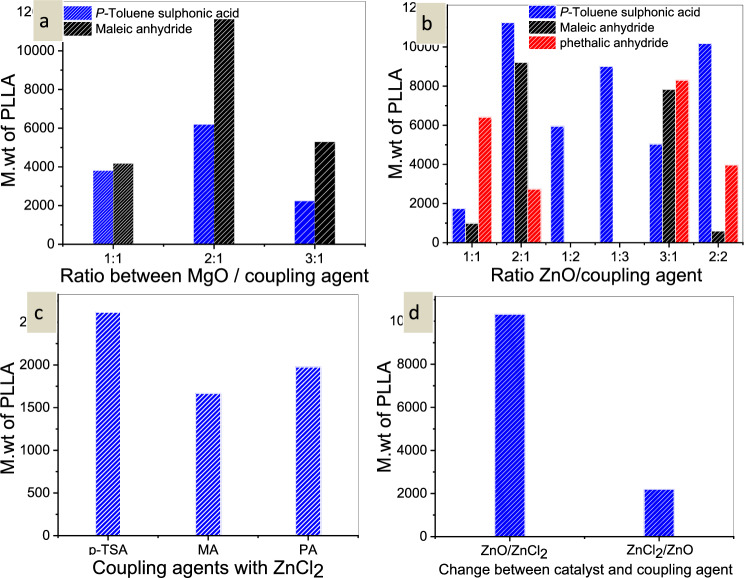
(**a**) Effect of the ratio between MgO/*p*-TSA and MgO/MA, (**b**) Effect of the ratio between ZnO and (*p*-TSA, MA, and PA), (**c**) effect of coupling agents with ZnCl_2_, and (**d**) Effect of change between catalyst and coupling agent in M.wt of PLLA.

To test the impact of coupling agents on the polymerization process, different coupling agents, such as *p*-Toluene sulphonic acid, maleic anhydride, and phthalic anhydride, were used with ZnO as a catalyst (Fig. 2). If *p*-Toluene sulphonic acid is used as the coupling agent and ZnO is used as the catalyst in a 2:1 ratio. This demonstrated the polymer’s large molecular weight. The molecular weight of the resulting polymer was enlarged by altering the coupling agent to maleic anhydride.

However, on substituting the coupling agent to Phthalic anhydride with 2:1. The molecular weight did not significantly rise, but it did increase when the ratio of ZnO to phthalic anhydride was changed to 3:1. Maleic anhydride was a successful coupling agent with ZnO in the polymerization of PLLA, according to the data.

In the presence of ZnCl_2_ as a catalyst, the impact of coupling agent type was investigated. Based on the outcomes, the molecular weight produced by *p*-toluene sulphonic acid was higher than that of the other coupling agents. Finally, *p*-toluene sulphonic acid was a superior coupling agent with metal chloride catalysts than maleic anhydride, which was good with metal oxide catalysts.

ZnO was more effective as a catalyst than a coupling agent, enhancing the formation of cyclic dimers, the first stage in the polymerization of PLLA (ring-opening), than the second step (ring-opening).

### Thermal gravimetric analysis (TGA)

#### TGA of the synthesized PLLA

The synthetic PLA’s thermal tolerance was observed y the loss in sample weight during heating at a programmed rate of 10 °C/min under a N_2_ gas stream. It is most likely that the loss of ester groups causes PLLA to degrade at temperatures between 300 and 400 °C^38^. According to the circumstances in Table 11, the TGA created by PLLA demonstrated reasonable thermal stability.

**Table 11:** Thermal stability data of PLLA samples.

No	Sample (catalyst used)	T_i_	T_onset_	T_max_
1	ZnO	114	213	290
2	ZnCl_2_	130	210	298
3	ZnO/*P*-TSA (2:1)	94	210	277
4	ZnO/*P*-TSA (2:2)	98	211	276
5	Sb_2_O_3_	100	200	340
6	SnCl_2_·2H_2_O (X/D)	108	235	340
7	MgO/MA (2:1)	97.7	208	348
8	Fe_2_O_3_	85.4	218	316
9	ZnO/ZnCl_2_ (2:2)	98	211	276

When PLLA was made using ZnO as a catalyst, the only actual decomposition began at 144 °C and reached its peak rapid weight loss of about 100% at 290 °C, but this occurred at 298 °C when ZnO was replaced with ZnCl_2_. When PLLA is synthesized with Sb_2_O_3_ or SnCl_2_._2_H_2_O, 100% of the weight is lost at 340 °C, but when Fe_2_O_3_ is used as the catalyst, both the thermal stability and the weight loss are maximized at 311 °C. Maximum thermal decomposition of PLLA occurred at 277 °C when ZnO was used as a catalyst and *p*-Toluene sulphonic acid were used as the coupling agent, but maximum thermal degradation occurred at 348 °C when MgO was used as the catalyst.

ZnO was used as a catalyst, and ZnCl_2_ as a coupling agent in the synthesis of PLLA, which produced 100% breakdown at 276 °C.

#### TGA of encapsulated isosorbide dinitrate in PLLA nanoparticles

**Figure 3:**
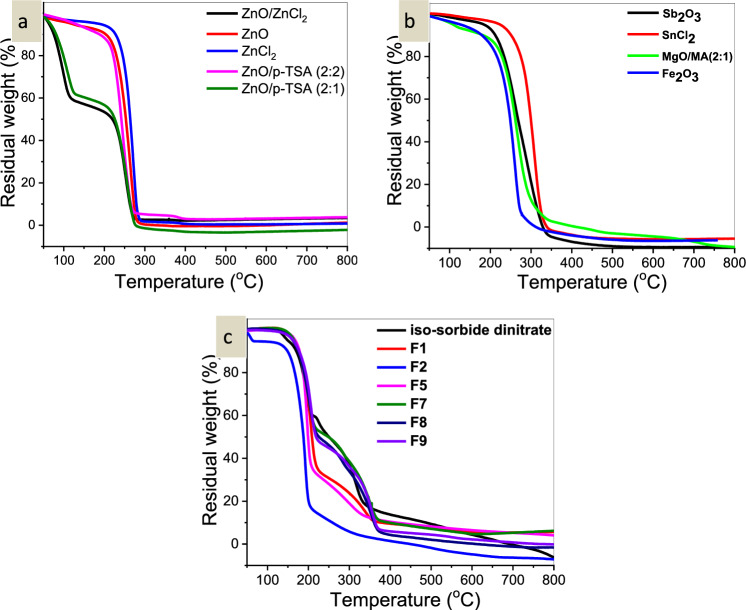
(**a**) TGA of PLLA samples synthesized by ZnO, ZnCl_2_, ZnO/ZnCl_2_, ZnO-*p*-toluene sulphonic acid (2:2), (2:1), (**b**) TGA of PLLA samples synthesized by Fe_2_O_3_, Sb_2_O_3_, SnCl_2_, and MgO/*p*-Toluene sulphonic acid (2:1), and (**c**) TGA of isosorbide dinitrate, F1, F2, F5, F7, F8, and F9.

Figure 3a, b depicts TGA of PLLA samples synthesized by ZnO, ZnCl_2_, ZnO/ZnCl_2_, ZnO-*p*-toluene sulphonic acid (2:2), (2:1), TGA of PLLA samples synthesized by Fe_2_O_3_, Sb_2_O_3_, SnCl_2_, and MgO/*p*-Toluene sulphonic acid (2:1). Also, TGA was used to investigate the thermal stability of encapsulated isosorbide dinitrate nanoparticles (Fig. 3c). According to the findings; nanoparticles are more thermally stable than individual PLLA. Additionally, it is easy to notice that isosorbide encapsulation significantly increases thermal stability (Table 12). And by adding more isosorbide dinitrate, the thermal stability was improved.

**Table 12:** Thermal stability data of encapsulated isosorbide dinitrate.

No.	Sample	T_i_	T_onset_	T_max_
1	Isosorbide dinitrate	127	352	615
2	F1	131	229	390
3	F2	114	272	428
4	F5	139	339	793
5	F7	136	235	674
6	F8	135	232	610
7	F9	137	236	707

### Differential scanning calorimetry (DSC)

#### DSC of the synthesized PLLA

**Figure 4:**
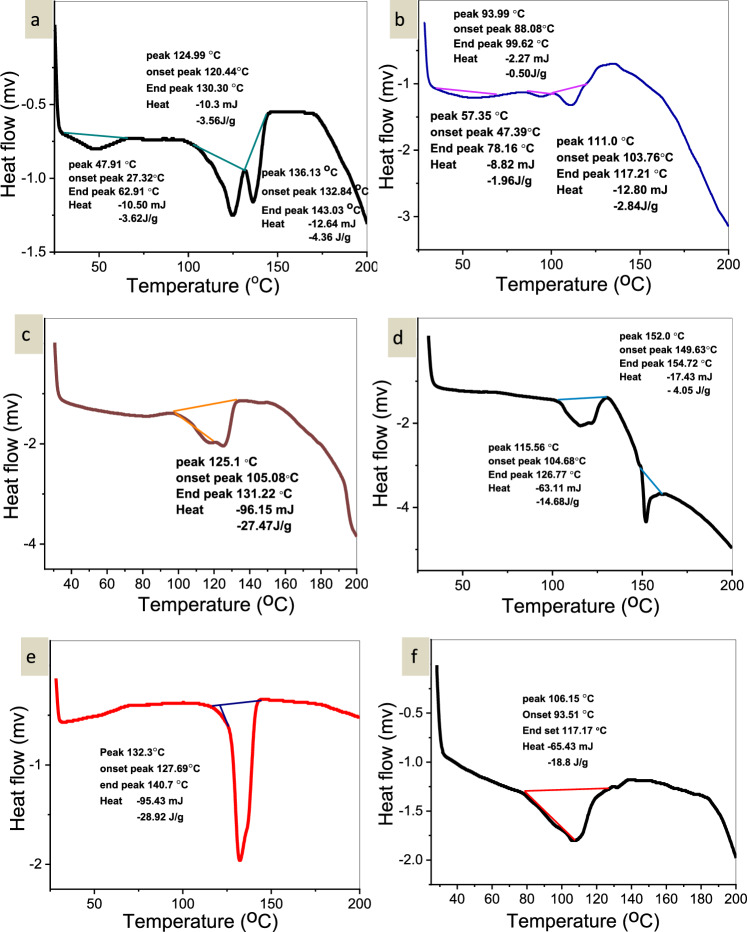
(**a**) DSC of PLLA with SnCl_2_.2H_2_O, (**b**) DSC of PLLA with MgO/MA (2:1), (**c**) DSC of PLLA with ZnO, (**d**) DSC of PLLA with ZnCl_2_, (**e**) DSC of PLLA with Antimony trioxide, (**f**) DSC of PLLA with ZnO/ ZnCl_2_ (2:2)**.**

**Figure 5:**
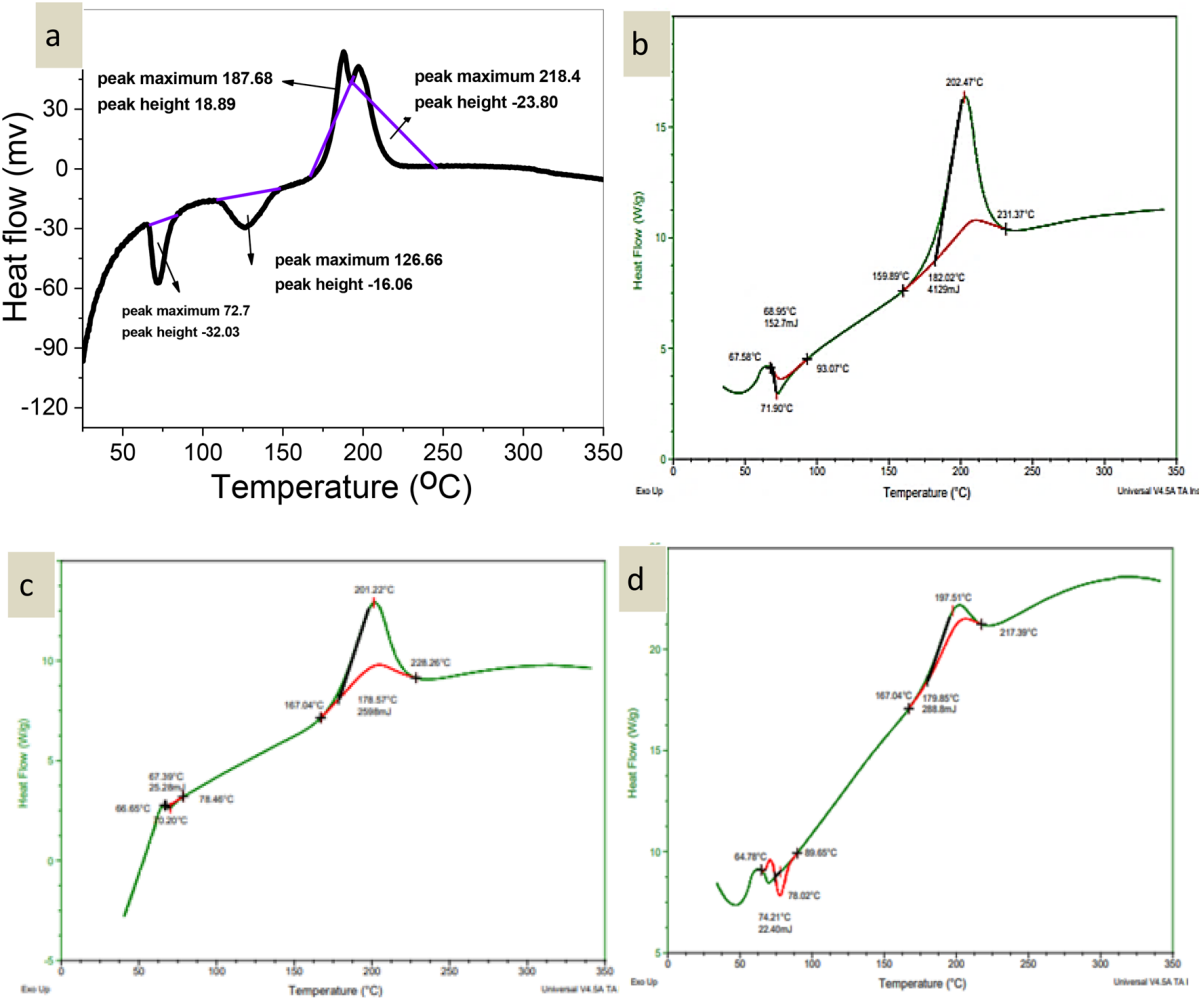
DSC of (**a**) isosorbide dinitrate, (**b**) DSC of F5, (**c**) F7, and (**d**) F9.

As can be seen from the DSC thermogram (Fig. 4), PLLA exhibited a transition temperature of 47 °C, with a corresponding melting temperature of 130 °C. In contrast, the endothermic peak of pure isosorbide dinitrate manifested itself at its crystalline melting point of 126.6 °C. The significant decrease in the melting temperature of isosorbide dinitrate in the nanoparticles (Fig. 5) indicates that it likely transforms from a crystalline to an amorphous phase during the manufacturing process or dispersion in the PLLA matrix. Detailed data on these temperature fluctuations can be found in Tables 13 and 14.

**Table 13:** DSC data of PLLA samples.

No.	Sample	Transition temperature	Melting temperature
1	SnCl_2_·2H_2_O	47	130
2	MgO/MA (2:1)	47	103
3	ZnO	46	125
4	ZnCl_2_	47	134
5	Se_2_O_3_	48	132
6	ZnO/ZnCl_2_ (2:2)	45	106

**Table 14:** DSC data of nanoparticles.

No.	Sample	Transition temperature	Melting temperature
1	Isosorbide dinitrate	72.2	218
2	F5	71.9	202.4
3	F7	67.3	201.2
5	F9	74.2	197.5

### X-ray diffraction analysis

**Figure 6:**
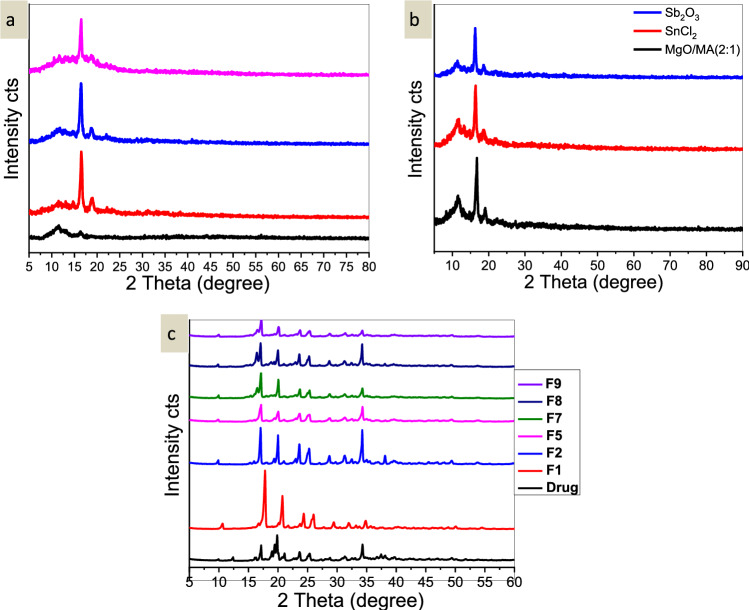
(**a**) XRD for PLLA by: black filled square: ZnO/*P*-toluene sulphonic acid (2:2), red filled square: ZnO (75 mg), blue filled square ZnCl_2_ (75 mg), and pink filled square: ZnO/ZnCl_2_ (2:2), (**b**) XRD for PLLA by: black filled square: MgO/Maleic anhydride (2:1), red filled square: SnCl_2_.2H_2_O (75 mg), and blue filled square: Sb_2_O_3_ (75 mg), and (**c**) XRD pattern of Isosorbide dinitrate, F1, F2, F5, F7, F8, and F9.

On various regions of every sample, X-ray diffraction was carried out. The XRD scans of the produced PLLA in the range of 2Ɵ = 5–80° were displayed in Fig. 6a, b. The primary diffraction peaks for PLLA at 2Ɵ = 16.40° were assigned to the orthorhombic (2–0–0) or octahedral (1–1–0) crystal planes, demonstrating that the PLA crystal had this characteristic orthorhombic structure. At 2Ɵ = 18.60°, a second, less intense peak that matched the crystal plane appeared (2–0–3). Additionally, this suggests that the crystal structure is orthorhombic. Other people also reported a similar outcome^39^. The peaks of PLLA were displaced to 2Ɵ = 17.3° and 20.2° by loading the drug, indicating that the encapsulation of the drug led to an increase in the crystallinity of PLLA, according to the XRD pattern of isosorbide dinitrate and encapsulated nanoparticles in Fig. 6c.

### Particle size and zeta potential analysis

Dynamic light scattering (DLS) was used to determine the particle size diameters of the nanoparticles encapsulating isosorbide dinitrate in the PLLA matrix, as well as their polydispersity index (PDI) and zeta potential, confirming their size and stability (Table 15). The observed particle size, ranging from 213 to 452 nm, is influenced by factors such as the type of surfactant, the polymer-to-drug ratio and the molecular weight of PLLA. A polydispersity index in the range of 0.38 to 1.00 indicates robust stability in water, while the zeta potential in the range of − 8.8 to − 22.4 supports the stability of the prepared particles in an aqueous environment. The negative zeta potentials are attributed to the presence of unbound carboxylic acid end groups on PLLA^40^.

**Table 15:** The particle size by number and volume, PDI, zeta potential, E.E, and D.L of prepared Nano-particles of isosorbide dinitrate encapsulated into PLLA.

Formula No.	Particle size (by intensity)	Particle size (by number)	PDI	Zeta potential	E.E (%)	D.L (%)
F1	452 ± 96.3	413 ± 100.2	0.593	− 8.8 ± 3.79	61.3	16.0
F2	217 ± 93.8	119 ± 49.7	0.192	− 8.69 ± 3.85	61.1	17.4
F5	310 ± 61.4	280 ± 64.2	0.620	− 17.0 ± 4.75	62	17.7
F7	291 ± 98.1	114 ± 72.2	0.380	− 13.1 ± 4.53	82.7	25.3
F8	213 ± 12.8	211 ± 26.2	1.00	− 13.1 ± 5.55	83.9	26.6
F9	254 ± 77.8	147 ± 77.8	0.404	− 22.4 ± 3.9	84.1	26.8

During emulsification, a substance that reduces surface tension is used to stabilize the droplets. Oil-in-water emulsions can be stabilized with various surfactants, including ionic and non-ionic polymer surfactants. Careful selection of surfactants is crucial to achieve particle sizes in the nano range. In addition to stabilizing the emulsions during the nano emulsion process, the type and concentration of surfactant play a crucial role in preventing particle aggregation and maintaining a low polydispersity index. In the context of pharmaceutical applications, toxicity and biocompatibility considerations of surfactants are essential.

### Effect of type of surfactant on particle size

PDI values and mean particle sizes for four samples made with various surfactants, including (Pluronic F88, Tetronic 1307, Tween 80, and PVA). In this study, it was discovered that Tetronic 1307, a surfactant, produces smaller nanoparticles than Pluronic F88, suggesting that it may be able to prevent the coalescence of oil globules. Tetronic molecules tend to align near the droplet’s surface, reducing the free energy at the interface between the two phases and preventing the droplets from coalescing. The emulsion generated with this drug was unstable, and phase separation occurred after a few hours of emulsification, producing polymer aggregates. Tween 80 and polyvinyl alcohol were found to be insufficient as surfactants.

#### Effect of the ratio between polymer and drug on particle size

It was discovered that the ratio of the polymer to the drug impacted particle size. While maintaining the same organic phase volume, increasing the polymer content causes the organic phase’s viscosity, which increases the viscous forces preventing droplet breakup and causing larger oil droplets to form, increasing particle size. Previous reports with comparable outcomes can be found in the literature^41^.

#### Effect of molecular weight of polymer on particle size

The relationship between the polymeric solution’s viscosity and molecular weight is straightforward. Similar experimental settings showed that a low-viscosity polymer solution could disperse in an external aqueous medium with small globules more effectively than a high-viscosity polymer solution, decreasing the size as the molecular weight increased^42^. The particle size was 291 nm for PLLA with a molecular weight of 24,165 Dalton but 213 nm for PLLA with a molecular weight of 11,051 Dalton.

#### Scanning electron microscope of encapsulated nanoparticles (SEM)

**Figure 7:**
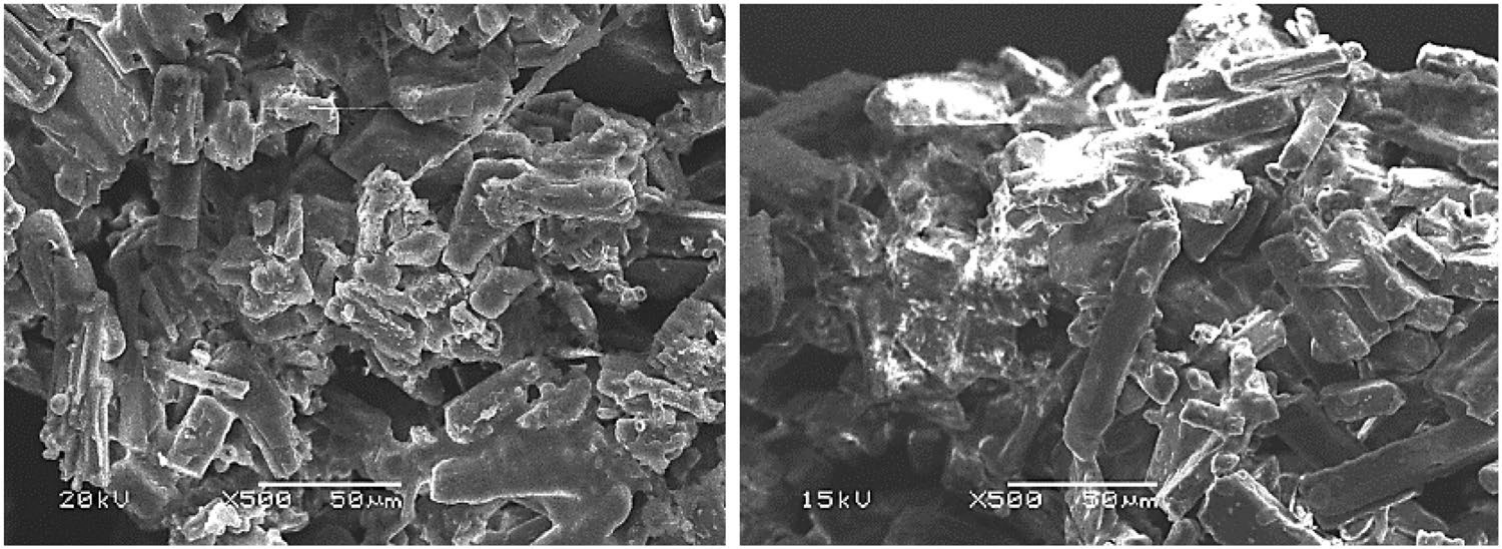
SEM image of F5.

The morphological analysis of the nanoparticles encapsulating isosorbide dinitrate was carried out using SEM, as shown in Fig. 7. The SEM images clearly show the existence of aggregated rod-like particles tightly packed in the sample. The observed aggregation of these rod-like structures is likely the result of interactions and integration of reactants with various surfactant aggregates^43^. Furthermore, using Zetasizer measurements, we found that the particle size ranges from 213 to 452 nm, providing valuable insights into the size distribution of the encapsulated nanoparticles. This comprehensive morphological and dimensional characterization improves the understanding of the nanostructure and enables a more sophisticated interpretation of the encapsulation process and its potential effects.

#### In vitro release of encapsulated isosorbide dinitrate in PLLA nanoparticles and kinetics of release

In vitro release data, monitored by UV as mentioned in the experimental section, were used to better understand the mechanism of isosorbide dinitrate release from PLLA nanoparticles. The E.E% and D.L% for the different formulas are shown in Table 15. For F9, the E.E% and D.L% were the highest among all formulas reaching 84.1 and 26.8% as Pluronic F88 was used for the preparation of samples F8 and F9. Pluronic-based drug delivery systems (DDS) are known for their remarkable ability to enhance the solubility of drugs. This property not only protects the drugs from potential hazards such as hydrolysis, enzymatic degradation or unwanted conjugation with biomolecules, but also helps to preserve their therapeutic efficacy. These DDS serve different purposes. Some focus on prolonged drug release, while others are designed for targeted delivery. For the latter, the aim is to exploit additional cellular release mechanisms or to ensure intracellular release in order to optimize the therapeutic effect. When developing an ideal nanoformulation, the main objective is to ensure that the drug is retained for the required period of time and released precisely at the site of action, while maintaining therapeutic concentrations for maximum efficacy.

The generated data were examined using various kinetic models previously described in the literature^44^. The release from the system is represented by the zero-order kinetic model, in which the release rate is independent of the concentration of the dissolved drug.

6 $${\text{Q}}_{{\text{t}}} = {\text{Q}}_{0} + {\text{K}}_{0} {\text{t}}$$

Q_0_ represents the initial dose, Q_t_ represents the cumulative amount of medication released at a time “t,” and K_0_ represents the zero-order release constant.

The identical approach was previously used^45^.

Where the release rate is concentration dependent is defined by the first-order release.

7 $${\text{Log Q}}_{{\text{t}}} = {\text{logQ}}_{0} + {\text{K}}_{{1}} {\text{t}}$$

where K_1_ is the first-order release constant.

Higuchi defined the release of drugs from an insoluble matrix as a square root of a time-dependent process based on the Fickian diffusion:

8 $${\text{Q}}_{{\text{t}}} = {\text{Q}}_{0} + {\text{KHt}}^{{{1}/{2}}} ,$$

where KH is the Higuchi constant.

The Hixson–Crowell cube root law defines the release from systems by dissolution where there is a change in the surface area and diameter of particles.

9 $$\sqrt[3]{{{\text{Qt}}}} - \sqrt[3]{{{\text{Q}}0}} = {\text{KHCt}}$$

where K_HC_ is the Hixson–Crowell constant.

Korsmeyer et al. derived a simple relationship, the Korsmeyer–Peppas model, which described drug release from a polymeric system.

10 $${\text{Qt}} = {\text{K}}_{{{\text{KPt}}}}^{{\text{n}}}$$

K_KP_ is the Korsmeyer–Peppas constant, and n is the release exponent describing the drug release mechanism^41^.

The parameters that offer the best fit between experimental findings and the nonlinear function were used in the modeling process. The model that best fits the release date was chosen based on the correlation coefficient (R^2^) in the models mentioned above. The model with the highest R^2^ value best fits the release data.

**Figure 8:**
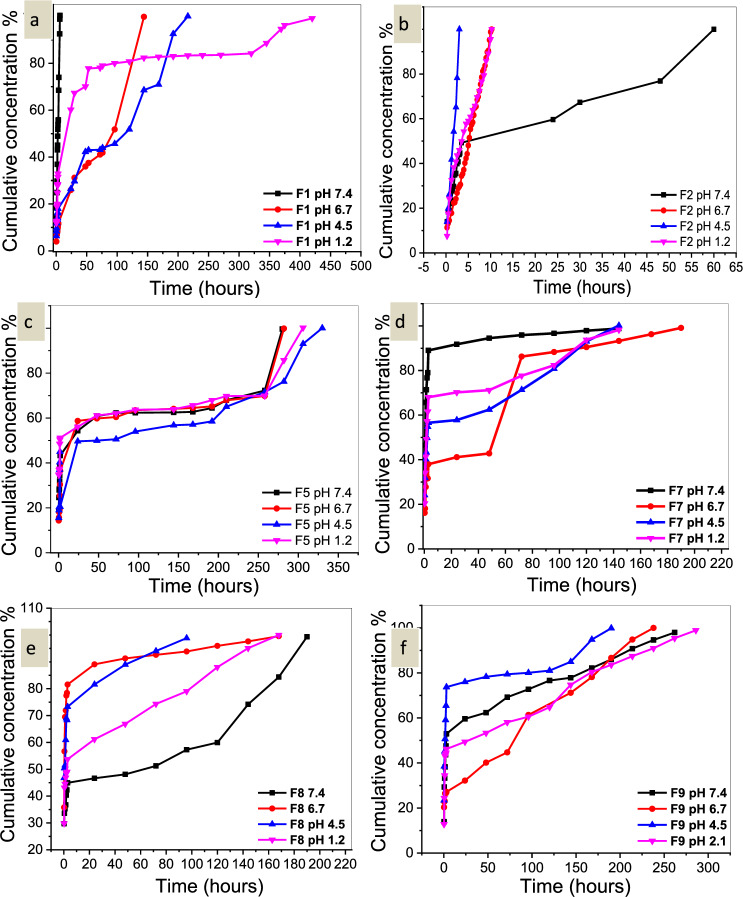
(**a**) Release study of isosorbide dinitrate from F1 in pH 7.4, 6.7, 4.5, and 2.1. (**b**) Release study of isosorbide dinitrate from F2 in pH 7.4, 6.7, 4.5, and 2.1. (**c**) Release study of isosorbide dinitrate from formula F5 in pH 7.4, 6.7, 4.5, and 1.2. (**d**) Release study of isosorbide dinitrate from formula F7 in pH 7.4, 6.7, 4.5, and 1.2. (**e**) Release study of isosorbide dinitrate from formula F8 in pH 7.4, 6.7, 4.5, and 1.2. (**f**) Release study of isosorbide dinitrate from formula F9 in pH 7.4, 6.7, 4.5, and 1.2.

The data show a two-stage release profile of isosorbide dinitrate (Fig. 8). During the first three hours of release, the isosorbide dinitrate bound to the surfaces of the nanoparticles was released. After 24 h, the amount of isosorbide dinitrate started to increase again, indicating that the drug contained in the PLLA NPs had started to diffuse. Since the carboxyl groups were less degraded in an acidic medium than in an alkaline medium, the effect of pH on the release behavior in fast formulas showed that the release was slower at lower pH values. This could be due to the fact that ionic interactions are relatively negligible in an acidic medium. The results of applying mathematical models to the release data (Table 16) showed that the Korsmeyer–Peppas semi-empirical model with n 0.3 provided an ideal fit. The goal of sustained release of isosorbide dinitrate from PLLA NPs was to improve patient compliance by achieving prolonged therapeutic drug concentration at the target site, reducing doses used, limiting side effects, reducing dosing frequency, modifying and improving isosorbide efficiency.

**Table 16:** The kinetics of release of isosorbide dinitrate from PLLA nanoparticles.

Formula No.		Zero-order		First order		Higuchi model		Korsmeyer-Peppas		Hixon-Crowell	
	pH	K_0_	R^2^	K_1_	R^2^	K_H_	R^2^	K	R^2^	K	R^2^
F1	7.4	0.0344	0.970	0.204	0.905	0.013	0.613	0.0208	0.924	0.021	0.441
F1	6.7	0.001	0.956	0.008	0.836	0.013	0.873	0.016	0.973	0.047	0.516
F1	4.5	0.0009	0.966	0.004	0.8144	0.005	0.938	0.0199	0.970	0.009	0.776
F1	1.2	0.0001	0.631	0.001	0.494	0.003	0.834	0.013	0.943	0.005	0.897
F2	7.4	0.002	0.839	0.01	0.615	0.083	0.729	0.019	0.914	0.023	0.609
F2	6.7	0.018	0.988	0.087	0.957	0.080	0.934	0.022	0.930	0.028	0.524
F2	4.5	0.066	0.631	0.295	0.964	0.148	0.945	0.044	0.978	0.156	0.402
F2	1.2	0.016	0.965	0.069	0.751	0.066	0.976	0.023	0.983	0.040	0.557
F5	7.4	0.014	0.773	0.001	0.667	0.001	0.847	0.012	0.913	0.009	0.508
F5	6.7	0.0001	0.742	0.002	0.621	0.0019	0.850	0.026	0.924	0.011	0.526
F5	4.5	0.011	0.841	0.001	0.680	0.001	0.839	0.021	0.906	0.012	0.369
F5	1.2	0.017	0.815	0.001	0.755	0.001	0.833	0.010	0.930	0.013	0.300
F7	7.4	0.0007	0.427	0.0017	0.311	0.001	0.558	0.027	0.815	0.004	0.804
F7	6.7	0.0008	0.874	0.003	0.750	0.010	0.920	0.021	0.955	0.018	0.758
F7	4.5	0.0008	0.864	0.0029	0.704	0.009	0.881	0.018	0.938	0.027	0.494
F7	1.2	0.0004	0.708	0.0026	0.508	0.0012	0.783	0.026	0.894	0.007	0.777
F8	7.4	0.0005	0.921	0.0021	0.914	0.0008	0.841	0.017	0.916	0.010	0.409
F8	6.7	0.0001	0.518	0.0021	0.389	0.0019	0.643	0.018	0.850	0.005	0.918
F8	4.5	0.0002	0.755	0.0027	0.664	0.002	0.861	0.010	0.966	0.007	0.844
F8	1.2	0.005	0.9229	0.0002	0.797	0.0012	0.958	0.018	0.968	0.013	0.559
F9	7.4	0.0001	0.836	0.001	0.611	0.0019	0.917	0.017	0.938	0.006	0.794
F9	6.7	0.0009	0.993	0.002	0.953	0.014	0.955	0.0203	0.945	0.027	0.518
F9	4.5	0.025	0.644	0.001	0.483	0.0012	0.7403	0.020	0.889	0.009	0.687
F9	1.2	0.0002	0.898	0.0018	0.663	0.003	0.917	0.020	0.918	0.010	0.664

#### Cytotoxicity of encapsulated isosorbide dinitrate nanoparticles

**Figure 9:**
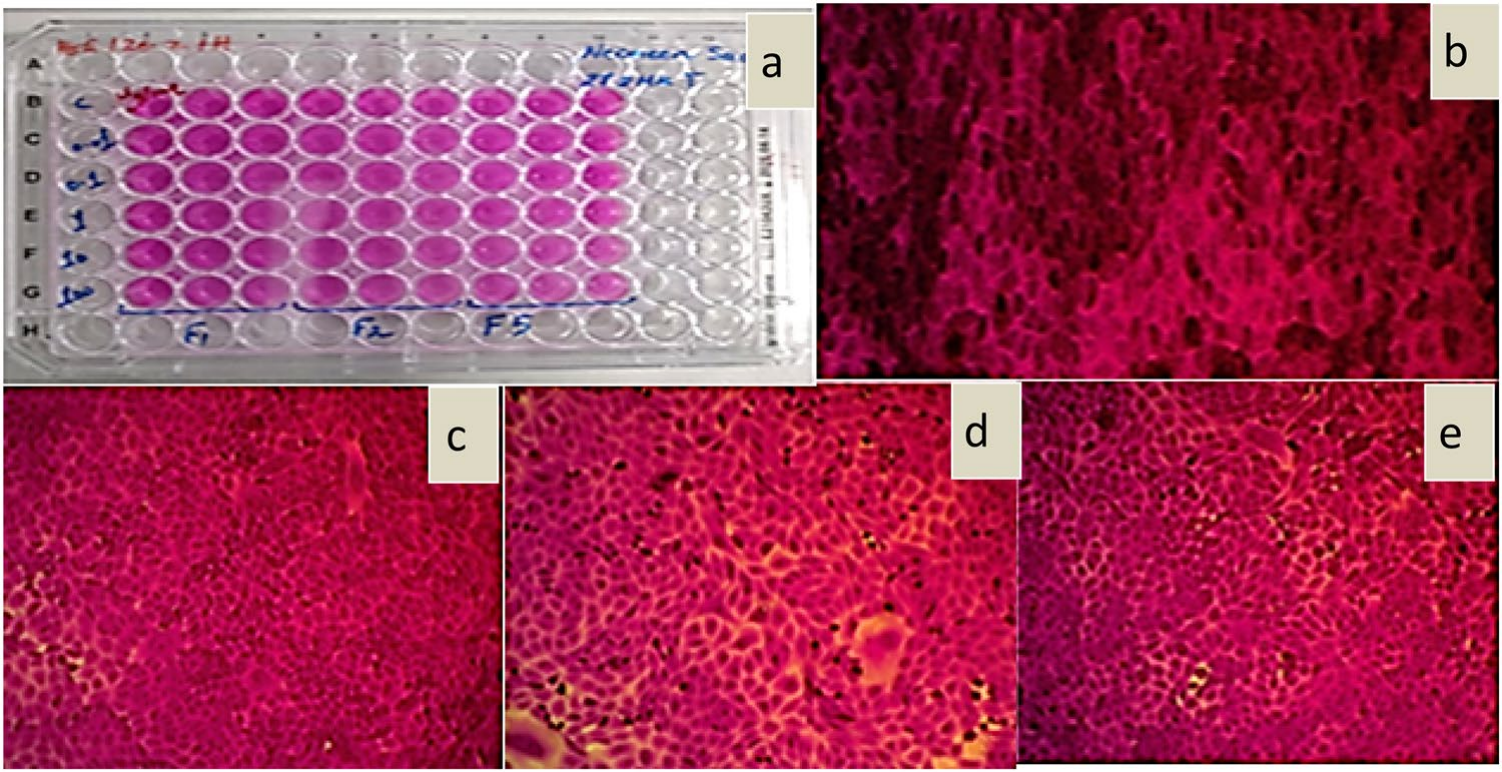
(**a**) Plate image, (**b**) Microscopic image of control, (**c**) Microscopic image of F1 concentration 1 µg/mL, (**d**) Microscopic image of F2 concentration 1 µg/mL, and (**e**) Microscopic image of F3 concentration 1 µg/mL.

**Figure 10:**
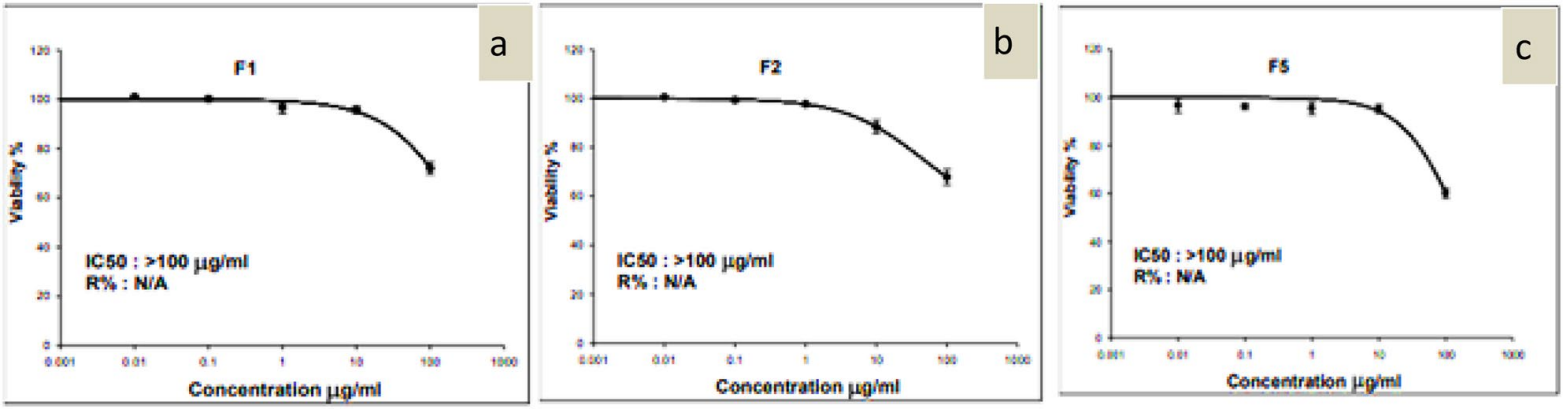
(**a**) Dose–response curve of F1, (**b**) dose–response curve of F2, and (**c**) dose–response curve of F5.

To ensure that isosorbide dinitrate is not hazardous to normal cells, the viability of the cells was assessed using the SRB assay on the (HSF) cell line. Figure 9 shows the morphology of the nanoparticle-treated cells to show that the nanoparticles did not impact the morphology of the cells. For each formula, the IC_50_ values were greater than 100, indicating the safety of the PLLA nanoparticles loaded with isosorbide dinitrate. Also, Fig. 10 shown Dose–response curve of F1, dose–response curve of F2, and dose–response curve of F5.

## Conclusion

The direct polycondensation method was used to synthesize PLLA. The FT-IR spectra showed no chemical interaction between the drug and polymer throughout nano encapsulation. The results demonstrated that PLLA yielded a greater M.wt using a solvent mixture than with xylene alone. The highest M.wt (25,114 Dalton) was obtained using antimony trioxide as a catalyst. The degree of polymerization increased as the catalyst concentration increased. Maleic anhydride was an effective coupling agent with ZnO in the polymerization of PLLA. *P*-toluene sulphonic acid was a more fabulous coupling agent with metal chloride catalysts than maleic anhydride, which was decent with metal oxide catalysts. XRD indicated that the encapsulation of the drug led to an increase in the crystallinity of PLLA. The results revealed that the release of the drug became slower in lower pHs, and the Korsmeyer-Peppas semi-empirical model with n 0.3 provided the greatest fit. The cytotoxicity of encapsulated isosorbide dinitrate nanoparticles showed that the IC_50_ values were greater than 100, representing the safety of the PLLA nanoparticles loaded with isosorbide dinitrate.

## Data Availability

Data are however available from the corresponding authors upon reasonable request.
